# A Camelid Anti-PrP Antibody Abrogates PrP^Sc^ Replication in Prion-Permissive Neuroblastoma Cell Lines

**DOI:** 10.1371/journal.pone.0009804

**Published:** 2010-03-22

**Authors:** Daryl Rhys Jones, William Alexander Taylor, Clive Bate, Monique David, Mourad Tayebi

**Affiliations:** 1 Department of Pathology & Infectious Diseases, Royal Veterinary College, Hertfordshire, United Kingdom; 2 Multiple Sclerosis Research Center of New York, New York, New York, United States of America; Ohio State University, United States of America

## Abstract

The development of antibodies effective in crossing the blood brain barrier (BBB), capable of accessing the cytosol of affected cells and with higher affinity for PrP^Sc^ would be of paramount importance in arresting disease progression in its late stage and treating individuals with prion diseases. Antibody-based therapy appears to be the most promising approach following the exciting report from White and colleagues, establishing the “proof-of-principle” for prion-immunotherapy. After passive transfer, anti-prion antibodies were shown to be very effective in curing peripheral but not central rodent prion disease, due to the fact that these anti-prion antibodies are relatively large molecules and cannot therefore cross the BBB. Here, we show that an anti-prion antibody derived from camel immunised with murine scrapie material adsorbed to immunomagnetic beads is able to prevent infection of susceptible N2a cells and cure chronically scrapie-infected neuroblastoma cultures. This antibody was also shown to transmigrate across the BBB and cross the plasma membrane of neurons to target cytosolic PrP^C^. In contrast, treatment with a conventional anti-prion antibody derived from mouse immunised with recombinant PrP protein was unable to prevent recurrence of PrP^Sc^ replication. Furthermore, our camelid antibody did not display any neurotoxic effects following treatment of susceptible N2a cells as evidenced by TUNEL staining. These findings demonstrate the potential use of anti-prion camelid antibodies for the treatment of prion and other related diseases via non-invasive means.

## Introduction

Prion diseases also known as transmissible spongiform encephalopathies (TSEs) are a group of closely related fatal transmissible neurodegenerative diseases that affect humans and animals [1]. Prion disorders are associated with conversion of the normal cellular prion protein (PrP^C^) into a disease-associated isoform, PrP^Sc^, that acquires increased β-sheet structure and detergent insolubility [2]. These diseases are characterised by the deposition and aggregation of proteins into highly stable, partially proteinase-resistant plaques and fibrils [3], leading to neuronal cell death and spongiform change of the brain parenchyma [4].

A number of drugs have been assessed for their efficacy in inhibiting prion replication, and these included polyanions [5], Iododoxorubicin, tetracycline [6], Congo red [7], polyene antibiotics [8], and quinacrine [9]. With the exception of an amphotericine analogue that had some effect on disease progression [10], these drugs have been shown to be ineffective in interacting with PrP^Sc^ *in vivo*. Moreover, their capacity to transmigrate across the BBB has not been established.

Following successful treatment of scrapie-susceptible neuroblastoma (N2a) cells [11], [12], [13] and scrapie-infected mice [14], immune-based therapy has become the most promising therapeutic approach for the treatment of prion diseases [15]. After the landmark report from White and colleagues [14], showing for the first time efficacy of anti-prion antibodies in treating animal prion-disease, other researchers have demonstrated the effectiveness of antibody-mediated therapy in delaying the onset of disease *in vivo* [16], [17], [18], [19], [20]. The antibody-mediated therapy approach was first investigated in scrapie susceptible neuroblastoma cells (N2a) [12], [13], then in transgenic mice with an anti-PrP antibody μ-chain [21]. This was followed by vaccinating scrapie-infected mice with rPrP [22], PrP peptides [23], and mucosal vaccination using live attenuated strain of Salmonella typhimurium expressing the mouse PrP gene [24], [25]. Crucially and for the first time, we have previously shown that passive transfer of anti-PrP monoclonal antibodies following inoculation of mice with scrapie-infected material via the intraperitoneal route led to inhibition of prion replication *in vivo* and animals survived throughout their life-span and remained free of detectable prion infection [14]. When the passive antibody transfer was started after onset of clinical signs of disease, all animals succumbed to prion diseases and were not rescued, indicating the inefficiency of these antibodies to transmigrate across the BBB.

We have previously raised a camelid anti-prion antibody, known as PrioV3, capable of crossing the BBB *in vitro* and *in vivo* via receptor-mediated transport (M. Tayebi & J. Greenwood, unpublished data; M. Tayebi et al, presented at the Neuroprion conference, Madrid, September 2008). PrioV3 displayed binding specificity for both PrP^C^ and PrP^Sc^ and was believed to bind PrP^C^ in the cytosol of neurons (Tayebi et al, submitted); In marked contrast, conventional anti-prion antibodies produced in mouse against similar target antigen were unable to enter the neuronal plasma membrane and instead decorated the cell membrane by staining surface PrP^C^ (Tayebi et al, submitted).

In this report, we show that PrioV3 anti-prion antibody was effective in crossing BBB, reduce peripheral prion replication in vivo and cured chronically scrapie-infected N2a cells and was also able to abolish prion replication. Finally, we also demonstrate here that PrioV3 antibody failed to trigger neurotoxic effects as previously shown with conventional anti-prion antibodies raised in mouse ([26], M. Tayebi and M. David, submitted).

The camelid anti-prion antibodies could potentially form an important tool for the neutralisation/clearance of prions in the cytosol of affected neurons and could be applied for the treatment of prion and other related protein-misfolding diseases.

## Results

### PrioV3 anti-prion antibody binds to prion proteins and peptide

**Figure 1:**
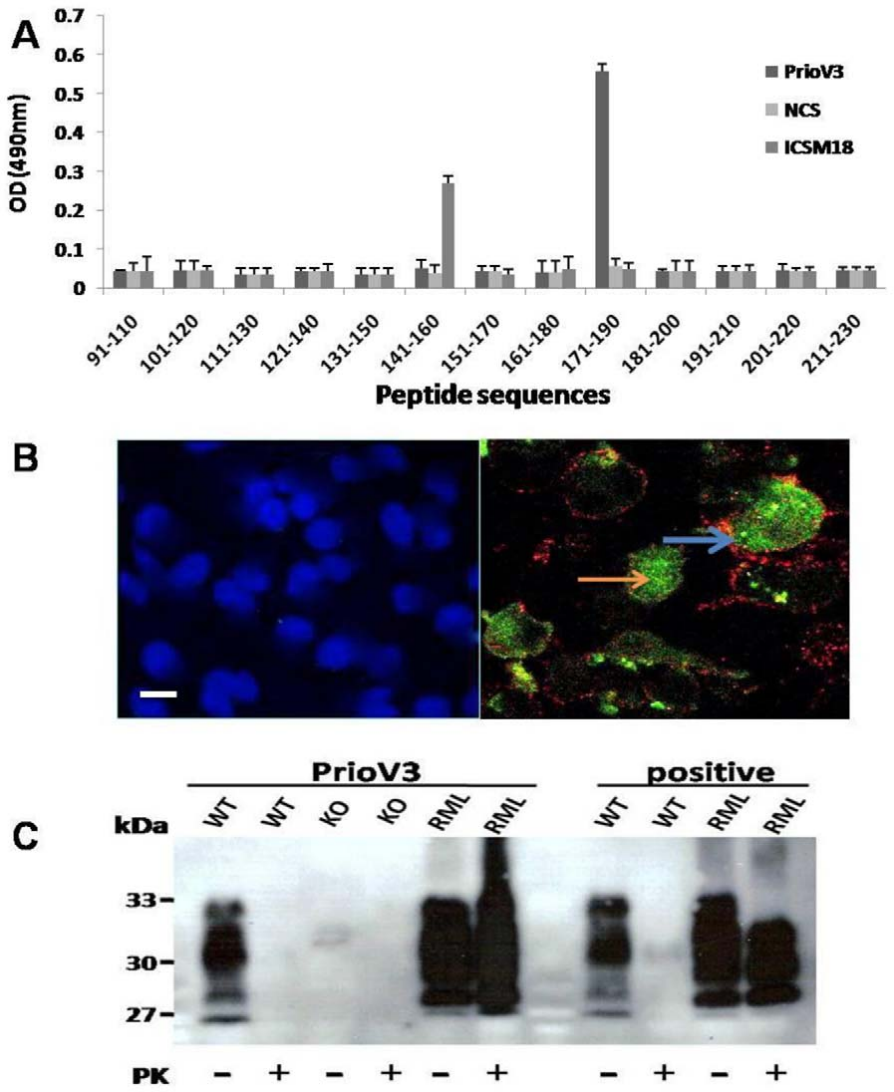
PrioV3 binds to both native and denatured prion proteins. (A) PrioV3 antibody was screened with 20-mer amino acid sequences spanning the 91–230 region of the prion protein and was shown to bind a region between 170 and 19. ICSM18 that binds to a region between 141 and 160 was used as positive control and normal camel serum (NCS) was used as negative control. Goat anti-llama IgG-HRP was used as secondary detection antibody. Anti-PrP responses were measured in peptide ELISA. Error bars represent the mean antibody level derived from n = 8 wells. (B) PrioV3 antibody bound to native PrP^C^ inside the cytoplasm of N2a cells (**orange arrow**), in contrast with ICSM35 that strictly stained cell membrane-bound PrP^C^ (**blue arrow**). Florescence microscopy was performed and images from each source [FITC (450–490 nm), Texas red (510–560 nm) and DAPI (330–380 nm)] were collected. As control, N2a cells were stained with the secondary anti-llama IgG (**green**) and anti-mouse IgG antibody (**red**), omitting the primary antibodies. Scale bar = 25 µm (C) PrP wild type, knock-out and RML-infected brain homogenate were digested with PK and tested for reactivity PrioV3. PrioV3 strongly bound both PrP^C^ and PrP^Sc^ in brain homogenates (pre- and post-PK). For comparison, ICSM35, raised in mouse (positive), was incubated with brain homogenates prepared from RML-infected and normal brain tissue with and with no PK digestion.

In order to confirm specificity to the prion protein and to determine its binding motif, PrioV3 anti-prion antibody was pepscanned (Figure 1A). 20-mer peptide sequences overlapping by 10 amino acids and spanning the 91–230 region of the truncated prion protein were used to epitope-map the PrioV3 antibody and was shown to bind to a linear motif on the C-terminal region of the protein, a sequence that lies between 171 and 190 (Figure 1A). This region was previously shown to bind to the so-called YYR motif used by Paramithiotis and colleagues to generate their PrP^Sc^-specific mAbs [27].

We have reported that PrP^C^ was widely expressed on the surface of N2a cells when probed with anti-prion mAbs raised against recombinant prion protein [11], [28]. Immunofluorescence analysis of PrioV3 antibody displayed strong binding to cellular prion protein expressed on the cell plasma membrane, suggesting its high affinity for PrP^C^ (Figure 1B). Furthermore, co-localisation studies have shown that binding with PrioV3 was also recognised in the cell cytoplasm by immunofluroscence following staining of intact N2a cells (non-fixed and non-permebealised), unlike ICSM35-stain that was strictly restricted to the plasma membrane (Figure 1B). Therefore, PrioV3 binds both surface and cytosolic PrP^C^ and recognizes residues 171 and 190 on the C-terminal region of the truncated PrP^27–30^.

To assess the ability of PrioV3 antibody to recognise PrP^Sc^, scrapie-infected (RML) or *Prn-p^−/−^* (KO) brain homogenate was treated with PK (QIAGEN) (Figure 1C). Western blot studies displayed similar binding pattern between PrioV3 and ICSM35; with several PrP^C^ bands from normal mice comprising one band at 27 kDa, two 29–32 kDa and 33–35 kDa, and one at 21–22 kDa. Following PK digestion, PrP^C^ was completely removed, whereas the 33–35 kDa form of PrP^Sc^ was shortened to 27–30 kDa, probably as a result of degradation of the amino-terminal segment of residues 23–90 analogous to hamster PrP^Sc^ [29]. PrioV3 antibody did not react with *Prn-p^−/−^* brain homogenates. Therefore, PrioV3 was shown to binds both PrP^C^ and PrP^Sc^ whether the protein is in native conformation or denatured.

### PrioV3 antibody is capable of crossing the BBB *in vitro* and *in vivo*

**Figure 2:**
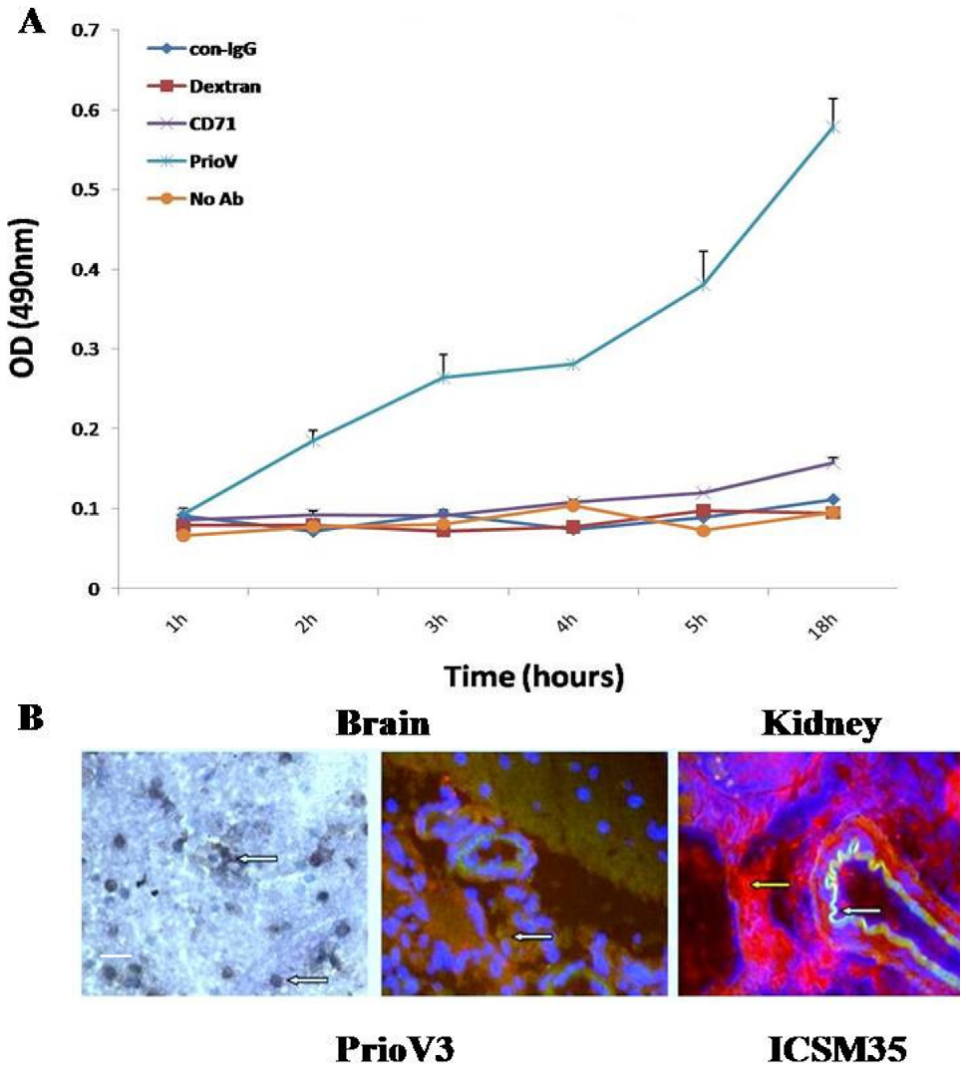
Assessment of PrioV transmigration across the BBB *in vitro* and *in vivo*. (A) BBB migration of PrioV3 antibody was assessed using GPNT rat and D3 human BBB models. PrioV was shown to cross both BBB cell lines efficiently as measured by ELISA, in contrast with CD71 antibody, Dextran and ICSM35 (con-IgG) that did not cross the BBB; (B) Immunohistochemistry and immunofluorescence stain of PrioV3 (**green**) in the brain parenchyma following intra-venous injection. PrioV3 antibody was injected i.v. to rats and brain sections were taken 4 h later to assess antibody migration across the BBB *in vivo* (white arrows). Co-injection with a conventional anti-prion IgG (**red**) displayed its inability to cross the BBB and staining remained in the kidney (yellow arrow). Scale bar = 5 µm.

Transport of PrioV3 and ICSM35 antibodies across the blood-brain barrier was assessed using GPNT rat [30] and HCMEC/D3 human [31] endothelial cells. PrioV3 was shown to cross both BBB cell lines efficiently as measured by ELISA (Figure 2A). In contrast, CD71 antibody, Dextran and ICSM35 antibody were unable to enter the BBB cell lines (Figure 2A). Furthermore, immunohistochemistry and immunofluorescence stain of the brain parenchyma of rats following intra-venous injection revealed that PrioV3 antibody was widely distributed, in contrast, ICSM35 did not display staining of the brain parenchyma but was seen in kidneys and liver of these rats (Figure 2B).

### PrioV3 antibody raised against PrP-Dynabeads inhibits accumulation of PrP^Sc^ in ScN2a Cells

We first assessed the efficiency of PrioV3, an anti-prion mAb antibody raised in camel against PrP-Dynabeads, as an inhibitor of prion replication in ScN2a cells.

ScN2a cells were cultured in triplicates and treated for 24 hours or 4 days at 37°C (5% CO_2_) with PrioV3 antibody. For the 4 day experiment, treatment was renewed daily in order to maintain levels of antibody above PrP^C^ levels, since PrP^C^ has a rapid turnover rate (half-time synthesis 0.1 hour vs. half-time degradation 5 hours) [32]. Normal camel serum (NCS) and no treatment were used as controls. The cells were then lysed and subsequently treated with PK prior to analysis of PrP^Sc^ levels by Sandwich ELISA and Western blot.

**Figure 3:**
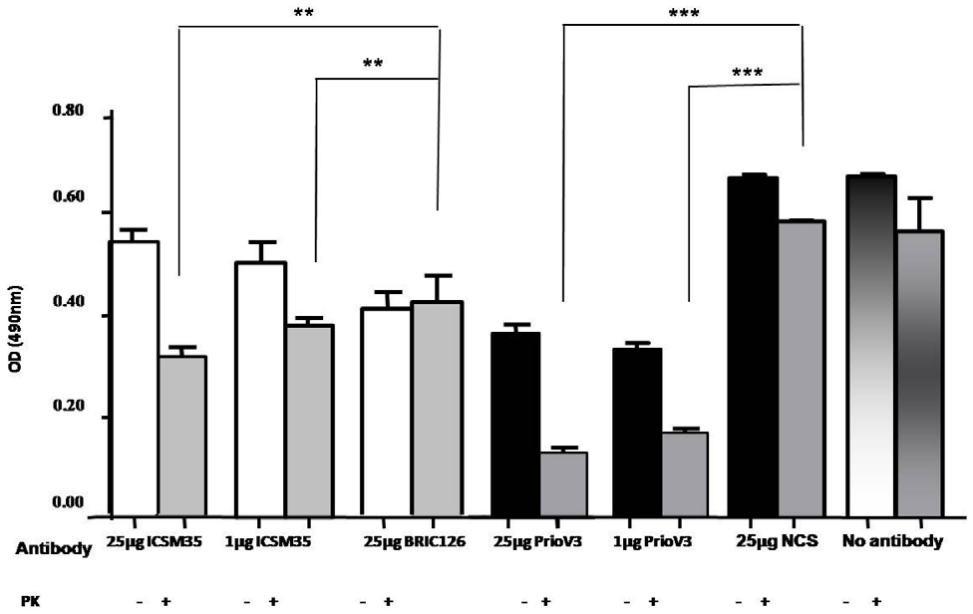
PrioV3 and ICSM35 antibodies inhibit accumulation of PrP^Sc^ in ScN2a cells. ScN2a cells were incubated for 24 hours at 37°C (5% CO_2_) with 1 or 25 µg PrioV3 and ICSM35. 25 µg NCS (Normal camel serum), and BRIC126-treated as well as untreated cells were also included. The cells were then lysed and subsequently treated with PK prior to analysis of PrP^Sc^ levels by Sandwich ELISA in triplicates. Both concentrations of PrioV3 used to treat ScN2a cells show a significant reduction in prion replication (P<0.001) when compared with the NCS control and the untreated cells. PrioV3 has also led to decreased levels of normal prion protein. ICSM35 led to a significant (P<0.001) reduction in prion replication compared to BRIC126-treated or untreated cells. Representative of three experiments.

For comparison, ScN2a cells were also treated with 1 or 25 µg ICSM35, an anti-prion mAb antibody raised against human recombinant β-PrP (Figure 3). ICSM35 binds residues 91 and 110 on the N-terminal region of the truncated PrP^27–30^ and was shown to be useful in delaying disease onset albeit leading to incomplete inhibition of PrP^Sc^ replication in the spleen of scrapie-infected mice [14], [33]. ICSM35 was also previously shown to disturb cholesterol homeostasis and alter cell signaling (M. Tayebi and M. David, submitted). An isotype matched control for ICSM35, BRIC126 was also used and untreated cells were included in the experiment (Figure 3).

As expected, ICSM35 led to a significant dose-dependent reduction in PrP^Sc^ accumulation after 24 hour treatment (Figure 3). When compared with PrioV3 antibody, ICSM35 was less potent in inhibiting PrP^Sc^ replication in ScN2a cells (Figure 3). ScN2a cells were treated with 1 µg or 25 µg PrioV3 antibody. Here, as little as 1 µg/ml of PrioV antibody abrogated PrP^Sc^ replication as demonstrated by Sandwich ELISA [(P<0.001), (Figure 3)]. Although 25 µg/ml of PrioV3 antibody led to a significant decrease of PrP^Sc^ as assessed by Sandwich ELISA, levels did not differ significantly from the 1 µg/ml of PrioV3 antibody treatment, suggesting the high potency of PrioV3 antibody in inhibiting PrP^Sc^ replication [(P<0.001), (Figure 3)].

We have previously shown that treatment of ovine PrP-inducible scrapie-infected Rov cells with a panel of the ICSM mAbs raised against both recombinant α-PrP and β-PrP led to significant reduction of PrP^Sc^ accumulation. It was determined that ICSM35 was the most effective in inhibiting PrP^Sc^ replication (x 100 to 1500) following treatment of Rov cells over a period 6 weeks [11]. This also suggests that PrioV3 antibody is a more potent inhibitor since it was shown to abrogate PrP^Sc^ replication after 24 hours only. In disagreement with previous work [11], PrioV3 antibody did alter PrP^C^ expression, independent of the dose or treatment length (Figure 3 and data not shown), indicating that the antibody has a direct neutralizing effect on PrP^C^ as well as PrP^Sc^. Of note, mAbs with higher affinity for PrP^C^ were shown to be more effective in reducing PrP^Sc^ replication in a dose dependent manner [12], [13], [14].

**Figure 4:**
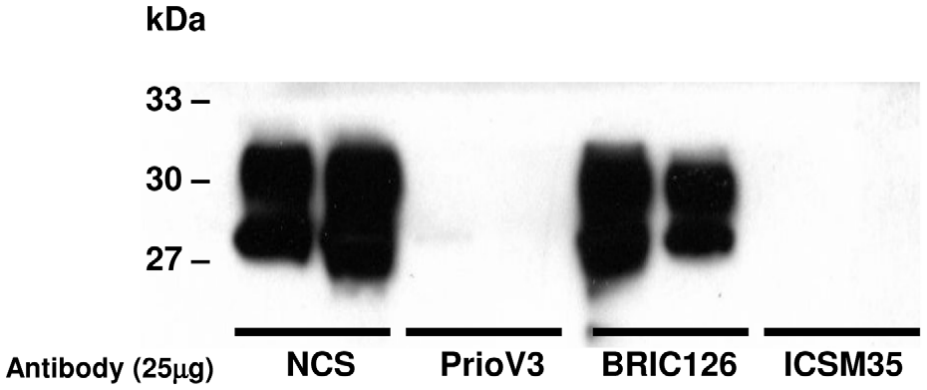
Long term treatment with PrioV3 or ICSM35 anti-prion antibodies abrogate PrP^Sc^ accumulation in ScN2a cells. ScN2a cells were incubated for 4 days at 37°C (5% CO_2_) with 25 µg ICSM35 or of PrioV3 and daily treatment renewal in tissue culture medium. BRIC126- and NCS-treated (Normal camel serum) were also included. The cells were then lysed and subsequently treated with PK prior to analysis of PrP^Sc^ levels by Western blot. Both PrioV3 and ICSM35 abrogated PrP^Sc^ replication when compared with the BRIC- and NCS-treated cells. Representative of three experiments.

ScN2a cells were also treated with 25 µg PrioV3 or ICSM35 over a period of 4 days (Figure 4) with daily treatment renewal in tissue culture medium. NCS and BRIC126 treatment were used as controls. Following treatment, cells were lysed and then PK treated as described above. Levels of PrP^Sc^ were then analysed in the cell lysates by Sandwich ELISA and Western blotting. Both PrioV3 antibody and ICSM35 completely abrogated PrP^Sc^ replication as shown by Western blot. NCS and BRIC126 had no effect on PrP^Sc^ levels (Figure 4).

It has previously been reported that treatment for three [13] or seven [12] days with anti-PrP^C^ antibodies abolished PrP^Sc^ replication as demonstrated by undetectable levels in Western blot, in agreement with the present findings.

**Figure 5:**
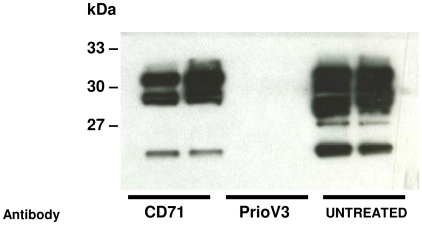
PrioV3 antibody inhibits PrP^Sc^ replication in spleens of antibody-treated mice. Weekly passive transfer of 2 mg PrioV3 antibody for 60 days induced substantial reduction in splenic PrP^Sc^ levels when treatment was started at day 10 p.i. CD71-treated as well as untreated mice did not inhibit splenic prion replication and this reduction was not observed.

In preliminary work, we assessed efficacy of our antibodies to inhibit prion replication *in vivo*. Here, mice were inoculated with RML scrapie brain homogenate and treated weekly with 2 mg of PrioV3 or isotype control antibody CD71 (IgG) from day 10 post inoculation (p.i.). Western blot analysis of PrP^Sc^ at 60 days p.i. showed a marked inhibition of PrP^Sc^ accumulation in the spleen (Figure 5 and data not shown). This pilot study confirms the inhibitory effects of PrioV3 on PrP^Sc^ inhibition *in vivo* and experiments are currently underway to assess the effects of PrioV3 on clinical disease in mice.

### Recurrence of PrP^Sc^ replication in ICSM35- but not PrioV3-treated cells

We have also assessed whether PrP^Sc^ accumulation recurs after treatment was ceased in PrioV3- and ICSM35-treated ScN2a cells.

**Figure 6:**
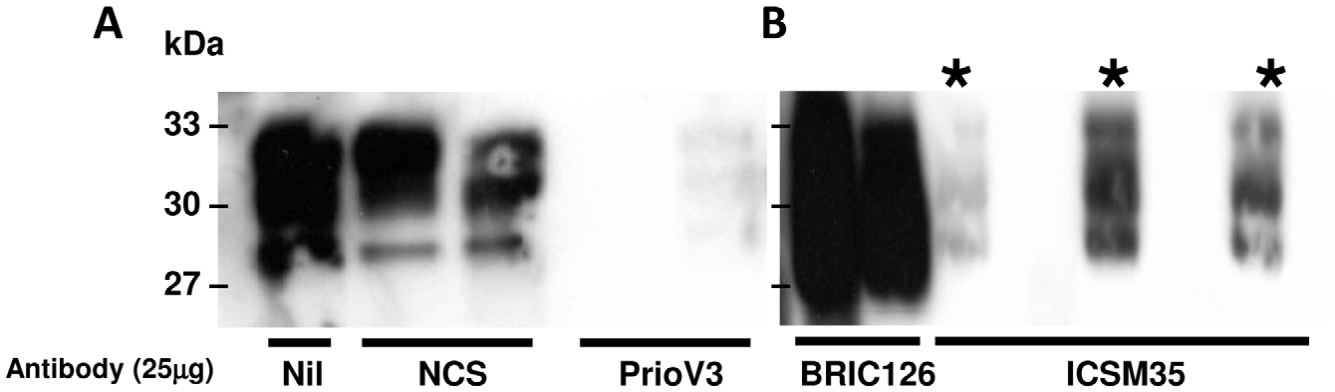
PrioV3 but not ICSM35 anti-prion antibody permanently inhibits accumulation of PrP^Sc^ in ScN2a cells following cessation of antibody treatment. ScN2a cells were incubated for 4 days at 37°C (5% CO_2_) with 25 µg of (A) PrioV3 and (B) ICSM35* and daily treatment renewal in tissue culture medium. NCS- (Normal camel serum) and BRIC126-treated cells were also included. The cells were then further grown for another three with antibody-free tissue culture medium. The cells were then lysed and subsequently treated with PK prior to analysis of PrP^Sc^ levels by Western blot. PrioV3 treatment led to permanent inhibition of PrP^Sc^ replication but ICSM35-treated cultures displayed de-novo replication of PrP^Sc^; both the BRIC- and NCS-treated cells showed high levels of PrP^Sc^. Representative of three experiments.

ScN2a cells were treated over a period of 4 days with daily treatment renewal of antibody in tissue culture medium with 25 µg of PrioV3 or NCS (Figure 6A) and ICSM35 or BRIC126 (Figure 6B). Untreated cells were also included in the experiment. Subsequently, cells were treated with antibody-free tissue culture medium for another 3 days and levels of PrP^Sc^ were assessed by Western blot following PK digestion of cell lysates.

Cell lysates assayed 3 days after removal of PrioV3 antibody failed to display detectable levels of PrP^Sc^ as demonstrated by Western blot (Figure 6A) in contrast to ICSM35-treated cells that displayed high expression levels of PrP^Sc^ (Figure 6B). Both untreated as well as cells treated with isotype-matched BRIC126 antibody showed high levels of PrP^Sc^ replication. Furthermore, 3 days after treatment was discontinued, cell lysates and supernatants were screened for the presence of PrioV3 or ICSM35 by ELISA. Both PrioV3 and ICSM35 were not detected indicating that PrioV3 inhibitory effect was not the result of residual antibody in the cells but to its potent effect on PrP^Sc^ (data not shown). Taken together, these results suggest that only PrioV3 antibody led to permanent depletion of PrP^Sc^ in chronically infected N2a cells.

### ICSM35 but not PrioV3 antibody leads to neurotoxicity

PrP^C^ appears to play a crucial role in the process leading to neuronal death [34] as neurotoxic effects associated with prion diseases are not triggered in the absence of PrP^C^ expression [35], [36]. Furthermore, a role in signal transduction for PrP^C^ was suggested as binding to anti-PrP antibodies led to PrP^C^-dependent Fyn activation [37] and modulation of calcium-dependent protein kinase C in mice [38]. Following a recent report by Solforosi and colleagues showing that cross-linking PrP^C^ with specific anti-prion antibodies leads to neuronal apoptosis *in vivo* [26], we have shown that ICSM35 antibody treatment of neurons alters cell membrane fluidity and leads to disruption of signalling proteins that reside in membrane domains (M. Tayebi and M. David, submitted).

To determine whether cross-linking PrP^C^ to PrioV3 antibody could produce neurotoxic effects *in vitro*, DNA fragmentation was evaluated in N2a cells following treatment with varying concentrations (0, 5 and 25 µg) of endotoxin-free PrioV3 or ICSM35 antibody.

**Figure 7:**
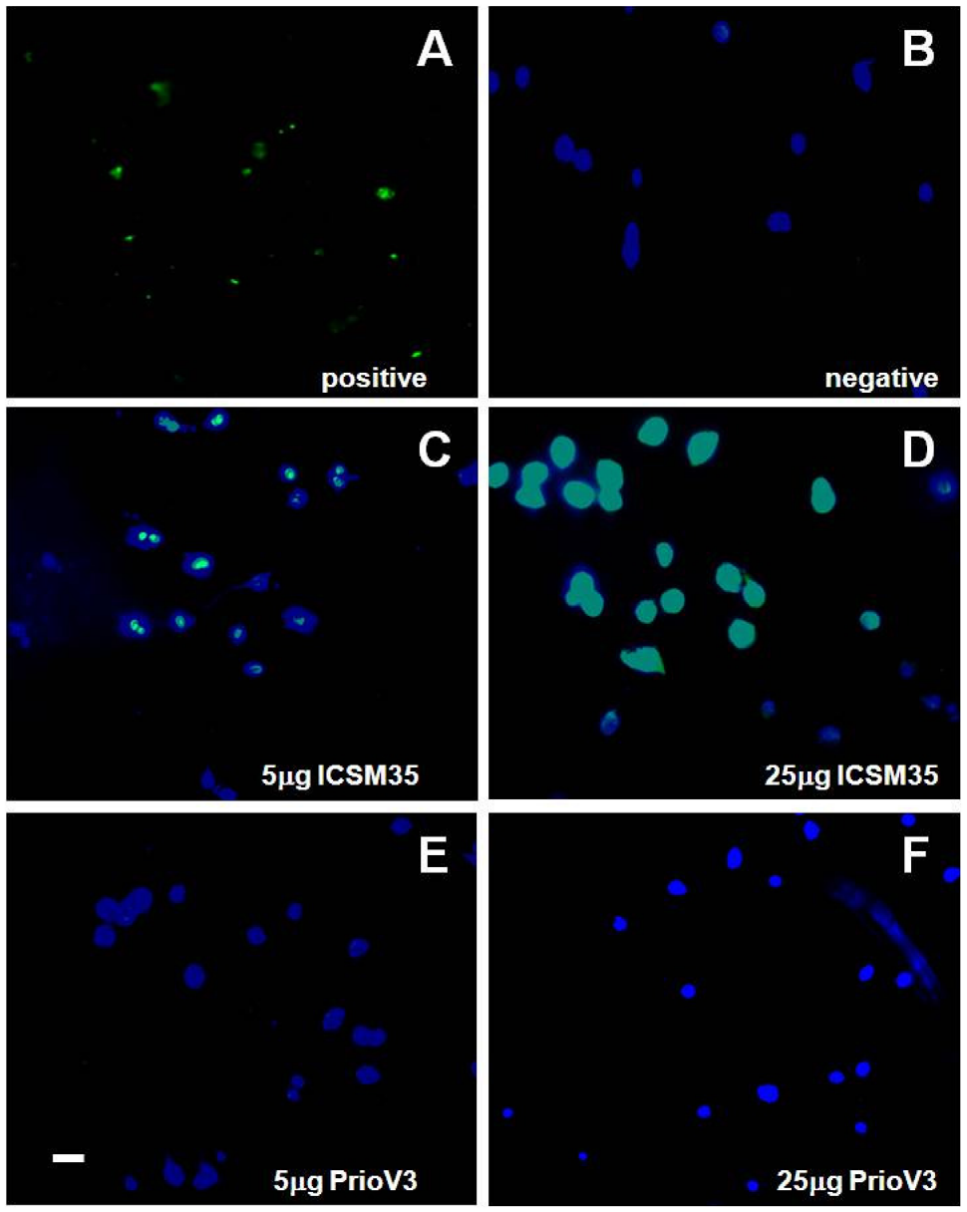
ICSM35 but not PrioV3 anti-prion antibody leads to neurotoxicity N2a cells following antibody treatment. N2a cells were assessed for neurotoxic effects by immunofluorescence imaging following treatment with PrioV3 or ICSM35. (A) Proprietary positive TUNEL stain and (B) antibody-free tissue culture medium were used as controls. Following treatment with 5 and 25 µg PrioV3 or ICSM35, TUNEL staining was only observed when cells were treated with ICSM35 (C & D). PrioV3 did not elicit DNA fragmentation and toxic effects were not observed (E & F). Cell nuclei were also stained with DAPI. Scale bar = 5 µm. Representative of three experiments.

This study showed that ICSM35 caused DNA fragmentation as shown by TUNEL stain in a dose-dependent manner (Figure 7C & D). In contrast, PrioV3 antibody failed to trigger any neurotoxic effect as demonstrated by TUNEL staining by immunofluorescence, even at the highest concentration (Figure 7E & F). Interestingly, only antibodies that recognize epitopes within the 91 to 110 region of PrP are capable of triggering neurotoxic-associated effects following cross-linking with PrP^C^ ([26], [M. Tayebi and M. David, submitted]). While PrioV3 antibody, that binds to a PrP sequence between 171 and 190, failed to elicit neurotoxic effects.

## Discussion

The conversion of normal prion protein (PrP^C^) into disease-associated PrP^Sc^ is a central event underlying the mechanisms of neuronal degeneration in prion diseases. Therefore, targeting both normal and disease-associated isoforms should form the basis for the development of effective therapeutic strategies for prion disorders.

Given that human prion diseases are normally diagnosed after onset of disease, it is crucial to develop compounds that can effectively arrest/reverse the disease process even after symptoms have began. Although, antibody-mediated therapy was shown to be effective for the treatment of rodent prion disease, intraperitoneal passive transfer of anti-PrP mAb did not have a protective effect following CNS invasion/inoculation of prions [14]. Due to their relatively large molecular mass, these anti-PrP mAb were unable to transfer into the CNS across the blood-brain barrier when administered peripherally.

Camelids generate functional antibodies consisting of only two heavy chains in comparison with the conventional four-chain antibodies; and differs from the corresponding regions of conventional antibodies in that they lack the CH1 domain [39]. Since they are devoid of the light chains, the antigen binding site of camelid antibodies is limited to a single domain. Camelid antibodies are tools of increasing interest for CNS therapy [40], [41], [42] and biophysical studies have revealed that they possess a number of unique features compared to those of conventional antibody fragments, notably smaller size (∼14 kDa), greater solubility and higher stability [40]. In that context, we have produced a camelid anti-prion antibody fragment, known as PrioV3, able to transmigrate across the BBB and cross the plasma membrane of neurons to target cytosolic PrP^C^ (Tayebi et al *submitted*).

In this report, we show that PrioV3 binds to both PrP^C^ and PrP^Sc^ and effectively abrogates prion replication in ScN2a cells, but it is unclear whether its inhibitory effects are due to recognition of normal and/or disease-associated isoform of prion protein. However, it was reported that PrP^C^ cleavage with phosphatidylinositol-specific phospholipase C from the cell surface [13] or stabilization with specific antibodies of PrP^C^ alone is sufficient to inhibit PrP^Sc^ replication [12], [13], [14]. Furthermore, Perrier and colleagues [43] demonstrated that anti-PrP antibodies block PrP^Sc^ replication in prion-infected cell cultures by accelerating PrP^C^ degradation.

We also show in this report that immunidetection of PrP^Sc^ from spleens of mice at 60 days p.i. revealed that treatment with PrioV3, but not with CD71 antibody markedly inhibited PrP^Sc^ accumulation when passive transfer started at day 10. This is in agreement with previous reports [14] demonstrating efficacy of a mouse mAb raised against alpha recombinant protein (i.e. ICSM18) in abrogating splenic prion replication following ip inoculation with RML. Of importance, mice treated with ICSM18 survived indefinitely [14].

Previous reports have shown that binding of anti-PrP antibodies to helix 1 region of PrP^C^, which is believed to play a crucial role in the conversion process [44], [45] inhibits PrP^Sc^ accumulation in ScN2a cells by preventing interaction of PrP^C^ with PrP^Sc^ [12], [13]. However, these antibodies were less efficient in clearing prion infection when used for the treatment of Rov cells [11], [12]. Thus, antibodies that bind to α-helix domain are able to abrogate prion replication in a murine neuroblastoma cell line (ScN2a; [12]), whereas, their ability to cure an ovine epithelial cell line (Rov cells) infected with prion was shown to be poor [11]. More importantly, ICSM35, which displays higher affinity for PrP^Sc^, also failed to completely rid scrapie-infected mice [14] and ScN2a cells (this study) of PrP^Sc^, but led to efficient prion inhibition in Rov cells [11]. Therefore, anti-PrP mAbs should be chosen on the basis that they selectively target species-specific prion strains.

The PrioV3 antibody was able to completely abrogate PrP^Sc^ replication in prion-permissive neuronal cultures even after treatment was ceased. This is in agreement with Enari and colleagues that demonstrated that ScN2a exposure to an anti-PrP^C^ mAb not only prevented infection but also cured chronically scrapie-infected cells after treatment was ceased [13]. In contrast, ICSM35 failed to prevent recurrence of the PrP^Sc^ replication in ICSM35-treated neuronal cultures [11]. Bioassay of infectivity from these lysates is in progress.

Mechanisms underlying differential outcome of PrP^Sc^ inhibition following treatment with anti-PrP antibodies remain unknown and clearly need to be further investigated, since antibodies thought to be effective in eradicating prion infection could in fact only suppress disease transiently. It could be speculated that variable levels of PrP^C^ expression in different cell lines/types would lead to diverse inhibitory effects [11], [13]. Further, differences in the binding affinities of antibodies used to inhibit PrP^Sc^ replication could also help to explain the difference in treatment outcome, given that PrP^C^ is ubiquitously expressed [46] and, in some cases, higher concentrations would be needed to achieve complete abrogation of prion replication. Binding specific motifs on the prion protein could also explain this dichotomy; for instance, antibodies that target the N-terminal but not the C-terminal region of the prion protein led to cell membrane disruption *in vitro* (M. Tayebi and M. David, submitted) and neurotoxic effects *in vivo* [26]. Whether the binding property of these antibodies to specific sequences is enough to explain these outcomes remain to be resolved. PrioV3 antibody could potentially turn out to be a more useful therapeutic tool for eradicating prion infection since it binds a more C-terminal region of the protein and targets both isoforms of the prion protein. In this case, PrioV3 antibody would be able to block PrP^C^ incorporation into propagating infectious agents and at the same time neutralize the infectious template, PrP^Sc^.

PrioV3 antibody displayed extensive and diffuse binding of PrP^C^ in the cytoplasm of cells without permebealization. PrioV3 ability to enter and target the cytosol of neurons remains unknown, but we speculate that this is due to the size of the antibody fragment (∼14 kDa) as ICSM35 (∼175 kDa) was unable to enter the cell.

Although many studies addressed the question of whether antibodies could inhibit PrP^Sc^ replication *in vitro* and *in vivo*, previous reports have demonstrated that cross-linking anti-prion antibodies with PrP^C^ leads to deleterious effects on neurons *in vivo* [26] and *in vitro* (M. Tayebi and M. David, submitted). Solforosi and co-workers used antibodies that specifically bind to epitopes within the N-terminal (95–105) and the C-terminal (133–157) regions of the prion protein. Interestingly, antibodies directed against codons within the 95–105 but not to the 133–157 the region led to apoptosis [26], while antibodies that target a more C-terminal region failed to display neurotoxic effects in prion infected mice and cells [18], [26]. Similarly, the current study showed that PrioV3 antibody which also binds to the C-terminal (71–190) region of the prion protein did not trigger neuronal apoptosis.

The mechanism(s) of prion-mediated neuronal degeneration are not fully understood but it is believed that this is caused by increased PrP^Sc^ neurotoxicity, so-called “gain of function hypothesis” as *prn*-*p* ^−/−^ mice displayed no obvious phenotype [47], or by loss of PrP^C^ function. It is likely that the apoptotic phenomenon observed after cross-linking of PrP^C^ with ‘toxic’ antibodies mimics the prion-mediated degenerative process and leads to antibody-mediated loss of function. The favourable efficiency and toxicity property observed for PrioV3 together with its ability to cross the BBB and to enter the plasma membrane of affected neurons makes it an attractive weapon in the fight against prion and other related diseases.

## Materials and Methods

### Production of anti-prion camelid antibodies

All procedures involving animals were carried out under a project and personal licence authority issued in accordance with The Animals (Scientific Procedures) Act 1986 and approval by the Royal Veterinary College Institutional Ethics Committee.

Preparation of Dynabead-adsorbed antigen for immunisation was performed as described [48]. Briefly, RML-infected brains from terminally ill scrapie-infected wild type FVB/N mice were used for the preparation of Dynabead-adsorbed antigen [48]. Sheep anti-mouse IgG-coupled immunomagnetic Dynabead particles (Invitrogen) were resuspended with an excess of IgG mAb anti-prion antibody (1–2 µg antibody/10^7^ Dynabeads). The Dynabeads/mAb complex was incubated overnight at 4°C then washed several times with PBS to remove excess mAb. 100 µl of 1% scrapie infected brain homogenate was incubated with 1×10^6^ mAb-coated Dynabeads for at least 2 hours at room temperature. The final complex, known as PrP-Dynabeads, was then rinsed and resuspended in PBS before immunisation.

Three adult male camels received six subcutaneous injections at weekly intervals of PrP-Dynabeads. For the first injection the antigen was mixed with an equal volume of Freund's Complete Adjuvant (FCA), and all subsequent boosts were with Freund's Incomplete Adjuvant (FIA). Forty-five days after the first injection, 50 ml of anticoagulated blood was collected to evaluate the immune response and used to isolate lymphocytes for mRNA extraction [49]. mRNA extraction was followed by cDNA preparation and the final PCR fragments were ligated into a phagemid vector as reported [49]. Ligated material was transformed in *Escherichia coli* cells and specific antibodies against prion proteins (PrioVs) were enriched by three consecutive rounds of *in vitro* selection. PrioV3 antibody was used in the current study.

ICSM anti-PrP mAbs (D-Gen) were produced as described [33]. ICSM 18, an IgG_1_, binds PrP^C^ strongly in its native state but weakly to native PrP^Sc^, and to both isoforms in Western blots. Its epitope lies between codons 144–156. ICSM 35, an IgG_2b_ binds both PrP^C^ and PrP^Sc^ from all species tested, whether the protein is in a native conformation or denatured. Its epitope lies between residues 91–110.

### Peptides and pepscan ELISA

20-mer peptides spanning the truncated prion protein and overlapping by 10 amino acids were made by automated solid phase step-wise synthesis using the Fmoc N terminal protection chemistry as described [48]. Briefly, peptides were cleaved from the solid phase and fully side-chain deprotected using trifluoroacetic acid with water and tri-isopropylsilane as scavengers. Cleaved peptides were precipitated and washed in ice-cold methyl tertiary butyl ether, dried, dissolved in suitable aqueous solvents and analysed by reverse phase HPLC and MALDI-TOF mass spectrometry.

High binding, 96 well plates (Greiner) were coated with 50 µl/well of a 10 µg/ml peptide solution in coating buffer (35 mM NaHCO3, 15 mM Na2CO3, pH 9.6). The plates were incubated for 1 hour at 37°C then washed 3 times with PBS-0.05% Tween 20, and then blocked with SuperBlock (SB; Pierce) for 1 hour at room temperature. After decanting the SB, 50 µl of PrioV3 antibody diluted in PBS-0.05% tween 20 was added and incubated for 1 hour at 37°C. The plates were then washed 3 times with PBS-0.05% tween and a 1/1000 dilution of horseradish-peroxidase (HRP) conjugated goat anti-llama IgG (Bethyl Laboratories) was added for 25 min at 37°C and the plates were again washed 4 times with PBS-0.05% tween. Finally, the plates were developed with OPD buffer (Sigma) until optimum development occurred, when the reaction was stopped with 3 M sulphuric acid prior to spectrophotometric reading at 490 nm.

### Tranmigration studies across the blood-brain barrier

Rat and human brain microvascular endothelial cell lines [30], [31] were used *in vitro* to assess trancytosis of both PrioV3 and ICSM35 antibodies. The brain microvascular endothelial cell lines were seeded on collagen-coated Falcon tissue culture inserts in the top chamber (1 µm pore size). The bottom chamber of the insert assembly contained appropriate medium (with no cells). The ability of PrioV3 antibody to transmigrate across the *in vitro* BBB model was assessed by adding 10 or 25 µg of the antibody into the upper compartment and determining the concentration in 200 µl aliquots from the bottom chamber at specified times using by ELISA.

In order to investigate whether PrioV3 retains the ability to cross the BBB *in vivo*, rats were injected in the tail vein or via intraventricular infusion with PrioV3 antibody or ICSM35. Four hours after injection, the animals were anesthetized. brain, liver and kidney were dissected and processed for histology.

### Cell culture

Mouse N2a neuroblastoma cultures were plated at 2–4×10^6^ in Opti-MEM medium [0.5% (W/V) glucose supplemented with 5% fetal bovine serum (FBS), 50 U/ml penicillin, 50 µg/ml streptomycin and 200 mM L-glutamine]. Cultures were maintained at 37°C in 5% CO_2_ with a change of medium every 48–72 hours.

### Immunofluorescence staining and imaging

For subsequent immunofluorescence staining with anti-prion antibodies (PrioV3 and ICSM35 antibodies), N2a cells were first seeded on glass coverslips in 35-mm dishes and grown to 50% confluence at 37°C in a humidified atmosphere of 5% CO2/95% on air. Coverslips were then rinsed three times in TBS (1 M tris-HCl, 1.5 M NaCl, pH 7.5) and 100 µl of blocking buffer [(1% (v/v) FBS, 1% BSA (w/v) in TBS] was added. The coverslips were incubated with 100 µl PrioV3 and/or ICSM35 for 1 hour at RT followed by the secondary antisera diluted in TBS [(anti-llama IgG FITC-conjugate, Bethyl; anti-mouse IgG Texas red-conjugate, Sigma)] for 1 hour at RT. After the final wash in TBS, the coverslips were mounted in fluorescence anti-fade solution (Invitrogen) and sealed with clear nail polish to prevent dehydration. 4, 6-dimidino-2-phenylindole (DAPI) (Sigma) was diluted to 2 µg/ml in fluorescence anti-fade solution for nuclear staining and added to coverslips.

Florescence microscopy was performed with a *Leica DM4000B* microscope. Images from each source [FITC (450–490 nm), Texas red (510–560 nm) and DAPI (330–380 nm)] were collected by a high resolution DC500 colour camera attached. All images are saved digitally using Leica's IM500 Image Manager Database software from the same field-of-view. Images were merged using Photoshop 6.0 (Adobe).

### Treatment of ScN2a with antibody

For subsequent treatment with anti-prion antibodies, ScN2a cells were plated using 6 or 24 well plates (Falcon) at 1×10^4^ cells/well and grown at 37°C in 5% CO_2_ for a minimum of 24 hours.

Appropriate antibody concentrations were added to 1×10^4^ ScN2a cells, and left to incubate for 24 hours or 4 days at 37°C (5% CO_2_). For the 4 day treatment, ScN2a cultures, antibody-containing tissue culture medium was added daily. The cells were then removed from the wells and centrifuged at 800 rpm for 5 minutes. The pellet was lysed with 50 µl lysis buffer followed by 1 µg/ml Proteinase K (PK; Roche) treatment for 25 minutes at 37°C. AEBSF protease inhibitor (Roche) was then added to inhibit PK activity. Cells were then stored at −20°C until further use. Levels of PrP^Sc^ in the culture were then analysed by Sandwich ELISA and Western blot.

To assess recurrence of PrP^Sc^ replication following removal of antibody, 1×10^4^ ScN2a cells were incubated for 4 days with daily replacement of antibody-containing tissue culture medium as described above. Following treatment stoppage, ScN2a cultures were continuously fed for another 3 days with tissue culture medium without antibody supplement. Cells were then treated with PK and analysed for the presence of PrP^Sc^ by Sandwich ELISA and Western blot.

### Protein extraction from cells

ScN2a cells were washed twice in PBS and homogenised in an extraction buffer containing 10 mM Tris-HCl, 100 mM NaCl, 10 mM EDTA, 0.5% Nonidet P-40, 0.5% sodium deoxycholate and 0.2% sodium dodecyl sulphate (SDS) at 10^6^ cells/ml. AEBSF protease inhibitor (Roche) was added to some cell extracts. Membranes were prepared by repeated passage with a Wheaton homogeniser; nuclei and large fragments were removed.

### Sandwich ELISA analysis

Medium binding, 96 well ELISA plates (Greiner) were coated with 50 µl of a 1 µg/ml ICSM18 antibody solution in coating buffer. The plates were incubated for 1 hour at 37°C then washed 3 times with PBS-0.05% tween, and then blocked with RF10 for 1 hour at room temperature. After decanting the RF10, cell lysates were diluted in PBS-0.05% tween with protease inhibitors (Roche Biochemicals) were added and incubated for 1 hour at 37°C. The plates were then washed 3 times with PBS-0.05% tween and a 1 µg/ml of biotinylated ICSM35 was added for 1 hour at 37°C and the plates were again washed 3 times with PBS-0.05% tween before a 1/1000 dilution of horseradish-peroxidase (HRP) conjugated anti-mouse IgG (Sigma) was added for 25 min at 37°C and the plates were again washed 4 times with PBS-0.05% tween. Finally the plates were developed with OPD buffer until optimum development occurred and the reaction was stopped with 3 M sulphuric acid prior to spectrophotometric reading at 490 nm.

### Western blot analysis

Samples to be electrophoresed were diluted 1∶1 in 40 µl sample buffer and boiled for 5 min in eppendorf tubes. The samples were spun for 5 seconds at 14.000 rpm in a microfuge before being loaded on the gel. The gels were electrophoresed at a constant voltage of 200 V for 1 hour. Following electrophoresis, gels were blotted onto PVDF (Invitrogen) at 18 V for 45 min. Following blotting, the membranes were rinsed in PBS-tween (0.05%) before being transferred to blocking solution for 60 min at room temperature. The membranes were again rinsed in PBS-tween (0.05%) to remove all traces of blocking solution. PrioV3, ICSM35 or ICSM18 antibody was added and incubated for 1 hour at room temperature. Following 4 washes of 5 min each, the membranes were then incubated in anti-mouse or anti-llama IgG HRP-conjugated antibody diluted at 1 in 20,000. The membranes were washed as above and developed using the Hybond-chemiluminescence (ECL) system (GE Healthcare), according to the manufacturer's instructions. Signal development times ranged from 1 second to 30 min.

### Assessment of neurotoxicity by TUNEL staining

Apoptosis was assessed by TUNEL staining using an apoptosis KIT according to manufacturing instructions (Calbiochem). DNA fragmentation was evaluated in ScN2a cells following treatment with varying concentrations of PRIOV3 and ICSM35. Briefly, cells were fixed with 50 µl of LEUCOPERM Reagent A (Serotec) for 10 minutes at room temperature. Cells were then washed with PBS and 100 µl of 5 x TdT Equilibrium Buffer was added (1∶5 TdT: H_2_O) and left to incubate for 10 min. After removing the solution, 30 µl of 1∶20 Fluorescein Labelling Reaction Mix: TdT Enzyme was added and incubated for 1 hour at 37°C. The slides were then washed 4 times with PBS for 15 minutes prior to adding 15 µl of DAPI nuclear stain (1∶600 DAPI: PBS). The cells were viewed with a LEICA DM4000B fluorescence microscope equipped with a Bio-Rad MRC1024 laser confocal scanning system and a LEICA DC500 camera. Images were layer-combined with Adobe Photoshop.

### Measuring splenic PrP^Sc^ levels following treatment of mice with PrioV3

Five FVB/N mice were inoculated via the intraperitoneal (i.p.) route with Rocky Mountain Laboratory (RML) scrapie brain homogenate derived from terminally scrapie-sick mice and treated with PrioV3 or isotype control antibody CD71 by a weekly i.p. injection (2 mg per injection) from 10 day post inoculation (p.i.).

### Statistical analysis

One-way ANOVA with Dunnett's post test was performed using GraphPad Prism version 5.00 for Windows, GraphPad Software, San Diego California USA, www.graphpad.com for statistical analysis.

## References

[1] Prusiner SB (1994). Biology and Genetics of Prion Diseases.. Annual Review of Microbiology.

[2] Prusiner SB (1998). Prions.. Proceedings of the National Academy of Sciences of the United States of America.

[3] Tayebi M, Bate C, Hawke S, Williams A (2007). A role for B lymphocytes in anti-infective prion therapies?. Expert Review of Anti-infective Therapy.

[4] Harris DA (1999). Cellular Biology of Prion Diseases.. Clin Microbiol Rev.

[5] Diringer H, Ehlers B (1991). Chemoprophylaxis of scrapie in mice.. J Gen Virol.

[6] Forloni G, Iussich S, Awan T, Colombo L, Angeretti N (2002). Tetracyclines affect prion infectivity.. Proc Natl Acad Sci U S A.

[7] Caughey B, Ernst D, Race RE (1993). Congo red inhibition of scrapie agent replication.. J Virol.

[8] Adjou KT, Privat N, Demart S, Deslys JP, Seman M (2000). MS-8209, an amphotericin B analogue, delays the appearance of spongiosis, astrogliosis and PrPres accumulation in the brain of scrapie-infected hamsters.. J Comp Pathol.

[9] Korth C, May BC, Cohen FE, Prusiner SB (2001). Acridine and phenothiazine derivatives as pharmacotherapeutics for prion disease.. Proc Natl Acad Sci U S A.

[10] Adjou KT, Demaimay R, Lasmezas C, Deslys JP, Seman M (1995). MS-8209, a new amphotericin B derivative, provides enhanced efficacy in delaying hamster scrapie.. Antimicrob Agents Chemother.

[11] Beringue V, Vilette D, Mallinson G, Archer F, Kaisar M (2004). PrPSc binding antibodies are potent inhibitors of prion replication in cell lines.. J Biol Chem.

[12] Peretz D, Williamson RA, Kaneko K, Vergara J, Leclerc E (2001). Antibodies inhibit prion propagation and clear cell cultures of prion infectivity.. Nature.

[13] Enari M, Flechsig E, Weissmann C (2001). Scrapie prion protein accumulation by scrapie-infected neuroblastoma cells abrogated by exposure to a prion protein antibody.. Proc Natl Acad Sci U S A.

[14] White AR, Enever P, Tayebi M, Mushens R, Linehan J (2003). Monoclonal antibodies inhibit prion replication and delay the development of prion disease.. Nature.

[15] Tayebi M, Hawke S (2006). Antibody-mediated neuronal apoptosis: therapeutic implications for prion diseases.. Immunol Lett.

[16] Pilon J, Loiacono C, Okeson D, Lund S, Vercauteren K (2007). Anti-prion activity generated by a novel vaccine formulation.. Neurosci Lett.

[17] Wuertzer CA, Sullivan MA, Qiu X, Federoff HJ (2008). CNS delivery of vectored prion-specific single-chain antibodies delays disease onset.. Mol Ther.

[18] Song CH, Furuoka H, Kim CL, Ogino M, Suzuki A (2008). Effect of intraventricular infusion of anti-prion protein monoclonal antibodies on disease progression in prion-infected mice.. J Gen Virol.

[19] Campana V, Zentilin L, Mirabile I, Kranjc A, Casanova P (2009). Development of antibody fragments for immunotherapy of prion diseases.. Biochem J.

[20] Tayebi M, Collinge J, Hawke S (2009). Unswitched immunoglobulin M response prolongs mouse survival in prion disease.. J Gen Virol.

[21] Heppner FL, Musahl C, Arrighi I, Klein MA, Rulicke T (2001). Prevention of scrapie pathogenesis by transgenic expression of anti-prion protein antibodies.. Science.

[22] Sigurdsson EM, Brown DR, Daniels M, Kascsak RJ, Kascsak R (2002). Immunization delays the onset of prion disease in mice.. Am J Pathol.

[23] Schwarz A, Kratke O, Burwinkel M, Riemer C, Schultz J (2003). Immunisation with a synthetic prion protein-derived peptide prolongs survival times of mice orally exposed to the scrapie agent.. Neurosci Lett.

[24] Goni F, Knudsen E, Schreiber F, Scholtzova H, Pankiewicz J (2005). Mucosal vaccination delays or prevents prion infection via an oral route.. Neuroscience.

[25] Goni F, Prelli F, Schreiber F, Scholtzova H, Chung E (2008). High titers of mucosal and systemic anti-PrP antibodies abrogate oral prion infection in mucosal-vaccinated mice.. Neuroscience.

[26] Solforosi L, Criado JR, McGavern DB, Wirz S, Sanchez-Alavez M (2004). Cross-linking cellular prion protein triggers neuronal apoptosis in vivo.. Science.

[27] Paramithiotis E, Pinard M, Lawton T, LaBoissiere S, Leathers VL (2003). A prion protein epitope selective for the pathologically misfolded conformation.. Nat Med.

[28] Khalili-Shirazi A, Quaratino S, Londei M, Summers L, Tayebi M (2005). Protein conformation significantly influences immune responses to prion protein.. J Immunol.

[29] Oesch B, Westaway D, Walchli M, McKinley MP, Kent SB (1985). A cellular gene encodes scrapie PrP 27–30 protein.. Cell.

[30] Regina A, Romero IA, Greenwood J, Adamson P, Bourre JM (1999). Dexamethasone regulation of P-glycoprotein activity in an immortalized rat brain endothelial cell line, GPNT.. J Neurochem.

[31] Weksler BB, Subileau EA, Perriere N, Charneau P, Holloway K (2005). Blood-brain barrier-specific properties of a human adult brain endothelial cell line.. FASEB J.

[32] Borchelt DR, Scott M, Taraboulos A, Stahl N, Prusiner SB (1990). Scrapie and cellular prion proteins differ in their kinetics of synthesis and topology in cultured cells.. J Cell Biol.

[33] Beringue V, Mallinson G, Kaisar M, Tayebi M, Sattar Z (2003). Regional heterogeneity of cellular prion protein isoforms in the mouse brain.. Brain.

[34] Chiesa R, Harris DA (2001). Prion diseases: what is the neurotoxic molecule?. Neurobiol Dis.

[35] Brandner S, Isenmann S, Raeber A, Fischer M, Sailer A (1996). Normal host prion protein necessary for scrapie-induced neurotoxicity.. Nature.

[36] Mallucci G, Dickinson A, Linehan J, Klohn PC, Brandner S (2003). Depleting neuronal PrP in prion infection prevents disease and reverses spongiosis.. Science.

[37] Mouillet-Richard S, Ermonval M, Chebassier C, Laplanche JL, Lehmann S (2000). Signal transduction through prion protein.. Science.

[38] Mazzoni IE, Ledebur HC, Paramithiotis E, Cashman N (2005). Lymphoid signal transduction mechanisms linked to cellular prion protein.. Biochem Cell Biol.

[39] Hamers-Casterman C, Atarhouch T, Muyldermans S, Robinson G, Hamers C (1993). Naturally occurring antibodies devoid of light chains.. Nature.

[40] Dumoulin M, Dobson CM (2004). Probing the origins, diagnosis and treatment of amyloid diseases using antibodies.. Biochimie.

[41] Abulrob A, Sprong H, Van Bergen en Henegouwen P, Stanimirovic D (2005). The blood-brain barrier transmigrating single domain antibody: mechanisms of transport and antigenic epitopes in human brain endothelial cells.. J Neurochem.

[42] Muruganandam A, Tanha J, Narang S, Stanimirovic D (2002). Selection of phage-displayed llama single-domain antibodies that transmigrate across human blood-brain barrier endothelium.. FASEB J.

[43] Perrier V, Solassol J, Crozet C, Frobert Y, Mourton-Gilles C (2004). Anti-PrP antibodies block PrPSc replication in prion-infected cell cultures by accelerating PrPC degradation.. J Neurochem.

[44] Morrissey MP, Shakhnovich EI (1999). Evidence for the role of PrP(C) helix 1 in the hydrophilic seeding of prion aggregates.. Proc Natl Acad Sci U S A.

[45] Speare JO, Rush TS, 3rd, Bloom ME, Caughey B (2003). The role of helix 1 aspartates and salt bridges in the stability and conversion of prion protein.. J Biol Chem.

[46] Bendheim PE, Brown HR, Rudelli RD, Scala LJ, Goller NL (1992). Nearly ubiquitous tissue distribution of the scrapie agent precursor protein.. Neurology.

[47] Bueler H, Fischer M, Lang Y, Bluethmann H, Lipp HP (1992). Normal development and behaviour of mice lacking the neuronal cell-surface PrP protein.. Nature.

[48] Tayebi M, Enever P, Sattar Z, Collinge J, Hawke S (2004). Disease-associated prion protein elicits immunoglobulin M responses in vivo.. Mol Med.

[49] Conrath KE, Lauwereys M, Galleni M, Matagne A, Frere JM (2001). Beta-lactamase inhibitors derived from single-domain antibody fragments elicited in the camelidae.. Antimicrob Agents Chemother.

